# Does depth of squat‐stand maneuver affect estimates of dynamic cerebral autoregulation?

**DOI:** 10.14814/phy2.14549

**Published:** 2020-08-18

**Authors:** Angus P. Batterham, Ronney B. Panerai, Thompson G. Robinson, Victoria J. Haunton

**Affiliations:** ^1^ Department of Cardiovascular Sciences University of Leicester Leicester UK; ^2^ Biomedical Research Unit in Cardiovascular Sciences National Institute for Health Research Clinical Sciences Wing Glenfield Hospital Leicester UK

**Keywords:** autoregulation index, cerebral hemodynamics, squat‐stand maneuvers, transcranial Doppler ultrasound, transfer function analysis

## Abstract

Repeated squat‐stand maneuvers (SSM) are an effective way of measuring dynamic cerebral autoregulation (dCA), but the depth of SSM required to improve dCA estimations has never been studied. We compared beat‐to‐beat cerebral hemodynamic parameters between maximal depth SSM (SSM_D_) and a shallower alternative (SSM_S_) in two age groups (younger [20–34 years] vs. older [50–71 years]) at a frequency of 0.05 Hz. Cerebral blood flow velocity, continuous blood pressure (BP) and end‐tidal CO_2_ (EtCO_2_) were measured using transcranial Doppler ultrasound, the Finometer device, and capnography, respectively. Coherence (at 0.05 Hz) was significantly higher in both SSM recordings compared to spontaneous BP oscillations at baseline standing (B_S_). Median (IQR) autoregulation index (ARI) was reduced during SSM_D_ (4.46 [4.03–5.22], *p* < .01) compared to SSM_S_ (5.96 [5.40–6.69]) and B_S_ (6.03 [5.20–6.49], *p* < .01) with similar relative differences also observed for phase (at 0.05 Hz). End‐tidal CO_2_ was increased in SSM_D_ (38.3 ± 3.7 mmHg, *p* < .01) compared to both SSM_S_ (36.6 ± 3.6 mmHg) and B_S_ (35.5 ± 3.2 mmHg). The older group demonstrated significantly lower ARI and phase estimates during SSM and found SSM_S_ more effortful than SSM_D_. In conclusion, both SSM_D_ and SSM_S_ are effective at estimating dCA, and dCA appears to be less efficient during maximal depth SSM compared to baseline rest or a shallower alternative.

## Graphical abstract

This study demonstrated the differences in dynamic cerebral autoregulation parameters between two depths of squat‐stand maneuvers (SSM) and two age groups. Maximal depth SSM yielded lower average autoregulation index (ARI) and phase compared to shallower depth SSM or baseline rest. Older subjects found shallower SSM more effortful than maximal depth SSM.

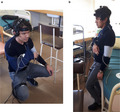

## 1 INTRODUCTION

Cerebral autoregulation (CA) describes the mechanism by which the cerebrovasculature is able to maintain a constant cerebral blood flow (CBF), despite changes in blood pressure (BP) (Van Beek, Claassen, Rikkert, & Jansen, 2008). Absent CA is characterized by a pressure‐passive relationship between mean arterial pressure (MAP) and CBF (Paulson, Strandgaard, & Edvinsson, 1990). Using the method of dynamic CA (dCA), first described by Aaslid et al (Aaslid, Lindegaard, Sorteberg, & Nornes, 1989), it is possible to investigate the relationship between pressure and flow in the cerebral circulation. This is achieved by quantifying the rapid modifications in CBF velocity (CBFv) in relation to manipulations in BP (Van Beek et al., 2008).

The dynamic relationship between changes in BP and subsequent effect on CBFv is often represented as a linear system, with BP acting as the “input” signal and CBFv as the “output” signal, allowing analysis of the temporal aspect of CA (Van Beek et al., 2008; Giller, 1990; Zhang, Zuckerman, Giller, & Levine, 1998).Transfer function analysis (TFA) allows the transfer of BP oscillations into CBF to be examined and quantified to give a measure of CA, using the parameters of gain, phase, and coherence (Zhang et al., 1998). Gain represents the dampening effect of CA on oscillations in BP, phase can be interpreted as the time delay for the autoregulatory response (Van Beek et al., 2008) and coherence indicates the degree of linearity between BP and CBFv, on a scale of zero to one (Giller, 1990; Panerai, Haunton, Hanby, Salinet, & Robinson, 2016). Coherence values above the 95% confidence limit are required to render reliable estimates of phase and gain (Claassen, Meel‐van den Abeelen, Simpson, & Panerai, 2016). A low coherence could be due to a number of factors: extraneous noise interrupting the signal (low signal‐to‐noise ratio [SNR]), more than one input influencing the output, an absence of relationship between input and output, or a nonlinear system (Zhang et al., 1998). The Autoregulation Index (ARI) quantifies the CA response to a step change in BP (Tiecks, Lam, Aaslid, & Newell, 1995). An increasing ARI indicates that the CBFv is correcting more quickly to a sudden change in BP and reflects a more effective CA response, with zero representing absent CA, and nine representing the best possible CA response (Tiecks et al., 1995).

In order to improve the coherence values, the SNR must be increased by inducing larger BP oscillations (Van Beek et al., 2008). Squat‐stand maneuvers (SSM) provide an effective, reproducible, and tolerable way of manipulating BP for the assessment of CA that can be used in healthy adult populations (Barnes, Ball, Haunton, Robinson, & Panerai, 2017, 2018; Claassen, Diaz‐Arrastia, Martin‐Cook, Levine, & Zhang, 2009; Claassen, Levine, & Zhang, 2009; Labrecque et al., 2017; Lewis et al., 2019; Smirl et al., 2014; Smirl, Hoffman, Tzeng, Hansen, & Ainslie, 2015; Zhang et al., 2009). The main drawback of SSM is the difficulty of their use in incapacitated subjects, people with mobility issues or those with cognitive impairment (Van Beek et al., 2008; Smirl et al., 2015). Despite the difficulty of applying SSM in some clinical conditions, a number of previous studies have already demonstrated its feasibility (Claassen, Diaz‐Arrastia, et al., 2009; Lewis et al., 2019; Smirl et al., 2014; Wright, Smirl, Bryk, & van Donkelaar, 2018).

Evidence from Claassen et al (Claassen, Diaz‐Arrastia, et al., 2009) and Barnes et al (Barnes et al., 2018) has addressed the questions of optimal frequency, and the number of SSM, respectively. However, there has been no research conducted in terms of the depth of SSM required to effectively measure CA using this technique. Previous investigations applying the SSM to study dCA, have used “deep” squatting, with participants lowering down until the thigh is parallel to the floor. However, if shallower SSM can provide accurate estimates of CA and are better tolerated by participants, it may make SSM available to patient populations previously excluded due to an inability to comply with study protocols. Studies using SSM for the assessment of CA have varied greatly in their methodologies regarding SSM depth, and evidence is lacking to suggest that these decisions have been made in a manner informed by research.

The influence of aging on dCA estimates derived from SSM has been studied with the classical SSM protocol (“deep” squatting) (Smirl et al., 2014; Smirl, Hoffman, Tzeng, Hansen, & Ainslie, 2016; Zhang et al., 2009), but not with the shallower version that we are proposing. Nevertheless, if shallower SSM are shown to be feasible, it is important to understand the influence of aging, and the ability of older subjects to comply with the protocol, before this new approach is extended to clinical applications.

The aims of this study, therefore, were to compare beat‐to‐beat CBFv, and other hemodynamic parameters, in younger and older groups of healthy participants during the performance of two SSM: one deep and one shallower alternative. Three main hypotheses were addressed: (a) Shallower SSM, measured as a 45‐degree flexion of the knee, have a similar effect on beat‐to‐beat hemodynamic parameters, TFA, and ARI estimates as deep SSM; (b) Aging does not affect differences between beat‐to‐beat hemodynamic parameters, TFA, and ARI estimates due to depth of the maneuver; and (c) Shallower SSM are more tolerable to healthy participants than deep SSM.

## 2 MATERIALS AND METHODS

### 2.1 Study subjects

Sixteen healthy younger (eight male, mean ± *SD* age 23.2 ± 3.4 years) and 18 healthy older (nine male, age 57.1 ± 5.5 years) participants were recruited from the University of Leicester. The study was carried out according to the latest approved protocol, the International Conference on Harmonisation‐Good Clinical Practice (ICH‐GCP), relevant regulations, and standard operating procedures, as well as in accordance with the Declaration of Helsinki. Ethical approval was obtained from the University of Leicester (Ref: 18199‐ab786‐ls:medicine, schoolof). All participants provided written, informed consent. Each subject was assigned a participant identification number, to which all their subsequent data were referred to thereafter, in accordance with the UK Data Protection Act and General Data Protection Regulations. Inclusion criteria for this study were as follows: staff or student at the University of Leicester, aged 18 years or older; capacity to consent to the study, willing to participate and able and willing to comply with all the study requirements. Female participants who were pregnant, lactating or planning pregnancy were excluded from the study, as well as participants with a diagnosis of atrial fibrillation, severe heart failure (ejection fraction <30%) or severe respiratory disease. One participant in the older group had a diagnosis of idiopathic hypertension and one participant in the older group had a history of postural hypotension.

### 2.2 Experimental protocol

All study participants attended a dedicated cardiovascular research laboratory, which was controlled at a temperature of 20–24°C and was free from distraction. Participants were asked to refrain from heavy meals, strenuous exercise, smoking, alcohol and caffeine for at least 4 hr prior to attending for assessment. Body‐mass index (BMI) was calculated using the weight and height from each subject.

**Figure 1:**
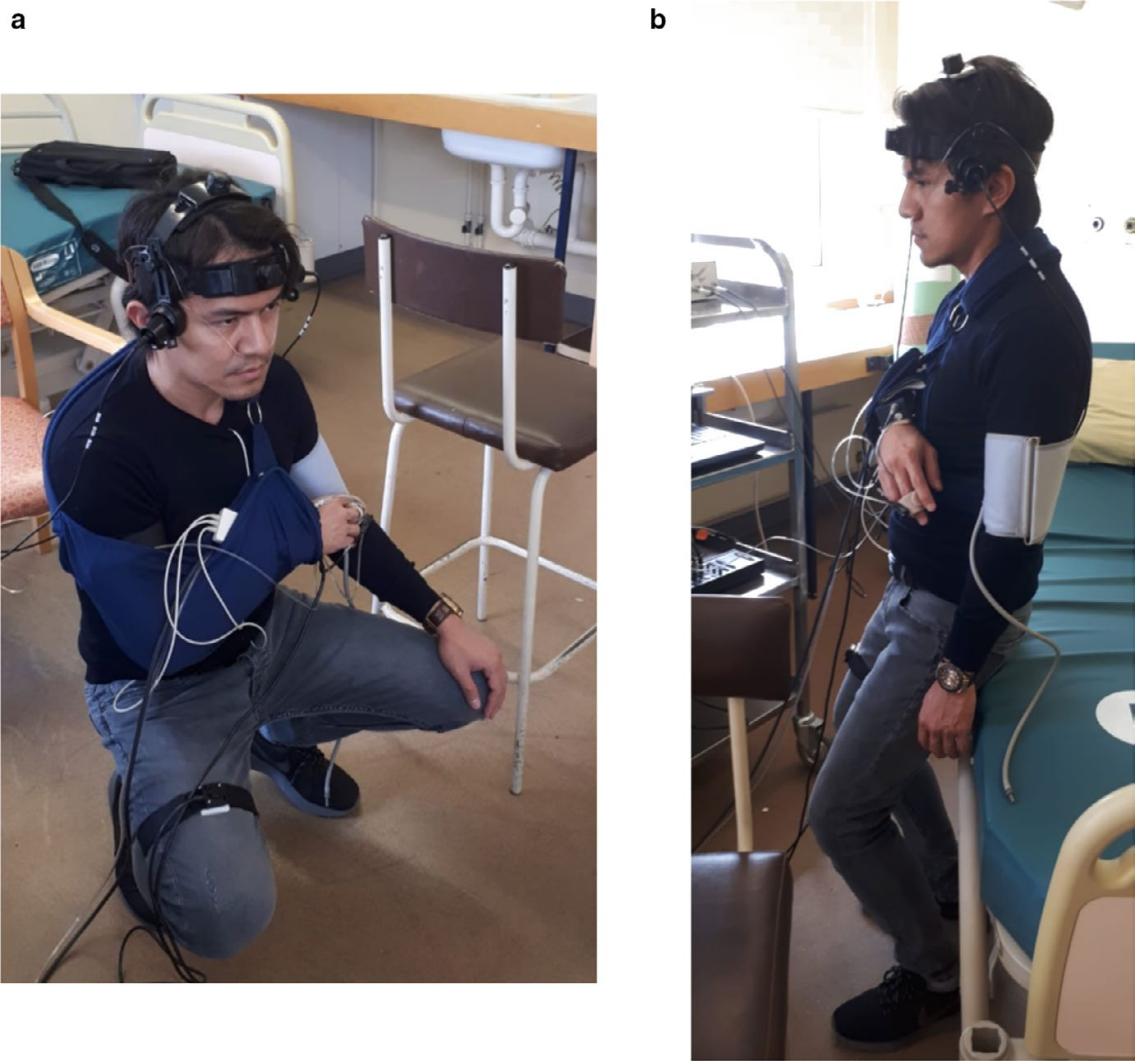
One participant during SSM_D_ (a) and SSM_s_ (b)

Once satisfactory signals had been obtained for all equipment, baseline brachial BP, HR, and EtCO_2_ measurements were taken, followed by three recordings: a 6‐minute baseline recording of the participant sitting quietly for 1 minute before standing up and remaining standing quietly for 5 minutes (“baseline standing”) with their eyes open; maximal comfortable depth SSM (SSM_D_); shallower depth SSM, measured as a 45 degree flexion of the knee (SSM_S_) (Figure 1). In all subjects, SSM_D_ were performed first, followed by SSM_S_.

During both SSM recordings, participants performed a maximum of 15 SSM at a frequency of 0.05 Hz (10 s standing, 10 s squatting), and a period of 90 s of standing quietly preceded and followed the SSM. Visual cues were provided via a computer program to guide the timings of the squat sequence. Participants were provided with a high chair on their left‐hand side, which they could touch lightly, to maintain balance, if needed. During SSM_S_, a bed was set at the correct height behind the participant to guide the depth of each squat, and they were instructed not to put any weight on the bed. Participants were asked to avoid a Valsalva‐like maneuver when in the squat positions and were allowed to rest for as long as needed between recordings, and before leaving the laboratory. Finally, the participants were asked to state which depth of SSM they found more effortful and to rate each depth on a level of exertion scale from 1 to 10 (1 = no exertion, 10 = exhaustion).

### 2.3 Instrumentation

Simultaneous bilateral insonation of the middle cerebral arteries (MCAs) through the temporal windows was performed using transcranial Doppler ultrasound (TCD, DWL Doppler box 10.5.1 Software) with 2MHz probes, held in place by a bespoke head frame. Heart rate was measured using three‐lead electrocardiogram. Beat‐to‐beat, noninvasive BP measurements were recorded using the Finometer cuff device, attached to the middle finger of the right hand (Finapres^®^ Medical Systems). The right arm was held steady using an arm sling, in order to minimize movement and keep the Finometer cuff at heart level. The PhysioCal mechanism was switched off during recordings to ensure a continuous BP trace, and was turned on again in between recordings to allow the device to calibrate. Brachial BP was also measured in between each recording using electrosphygmomanometry (UA 767 BP monitor) to calibrate the recordings from the Finometer. Respiratory rate and end‐tidal CO_2_ (EtCO_2_) were monitored using nasal cannulae (Salter Labs, ref 4000) attached to a capnograph (Capnocheck Plus). A tilt‐sensor attached to the participant's right thigh measured the depth and speed of squat (QG‐KI‐090AI‐K, DIS Sensors). All signals were simultaneously recorded onto the Physiological Data Acquisition System (PHYSIDAS, Leicester Medical Physics Department), at a sampling rate of 500 samples/s, for subsequent offline analysis.

### 2.4 Data analysis

Data were visually inspected using a purpose‐designed software program written in Fortran. Narrow spikes (<100 ms) and artefacts in the recordings were manually removed by linear interpolation. The CBFv signal was passed through a median filter and all recordings were filtered in the forward and reverse direction using an eighth‐order Butterworth low‐pass filter with a 20 Hz cut‐off frequency. The Finometer readings were calibrated to the brachial BP recordings. The beginning and end of each cardiac cycle were marked from the ECG signal, and mean values for BP, HR, and CBFv were calculated for every heartbeat. Beat‐to‐beat parameters were interpolated with a third‐order polynomial and then resampled at 5 Hz to produce signals with a uniform time base (Katsogridakis et al., 2013). For the SSM recordings, a custom‐made program marked the files under visual inspection just prior to each step change in BP, guided by the tilt sensor output, thereby producing 15 marks within each file. Intrafile coherent averaging produced a mean and standard deviation for each hemodynamic parameter per subject, per file, as a function of time.

Transfer function analysis (TFA) was performed in accordance with recent White Paper guidelines (Claassen et al., 2016), using BP as the input signal and changes in CBFv as the output. The fast Fourier transform (FFT) approach combined with Welch's method (Welch, 1967) provided frequency dependent estimates of phase, gain and coherence. These TFA parameters were extracted at the frequency of squatting (0.05 Hz) and also averaged across the very low frequency (VLF, 0.02–0.07 Hz) range (Claassen et al., 2016; Zhang et al., 1998).

CBFv response to a step change in BP was derived using the inverse FFT, which was compared to the 10 template curves proposed by Tiecks et al (Tiecks et al., 1995). The best fit curve, corresponding to the minimum mean square error, determined the ARI value. The values of ARI were accepted if the coherence function in the 0.15–0.25 Hz frequency interval was above its 95% confidence limit, and the normalized mean square error (NMSE) for fitting the Tiecks model was below the threshold of 0.3, which indicates physiological plausibility (Panerai et al., 2016). In the case of the standing baseline recording containing the sit‐to‐standing transition, the first 70 seconds of the recording were excluded from the TFA and ARI estimates.

### 2.5 Statistical analysis

Data are provided as means and standard deviation (*SD*) when normally distributed, and medians and interquartile ranges (IQR) when not normally distributed. Shapiro–Wilk W statistic was used to determine normality. Comparison between hemispheres for each variable was performed using Student's *t* tests and Wilcoxon ranksum tests. In the absence of any hemispheric differences, values were averaged across the right and left hemispheres. Hemodynamic parameters were compared between baseline recordings and SSM depths using one‐way repeated‐measures ANOVA if normally distributed and the nonparametric equivalent, Friedman test, if the data were skewed. If corresponding *F*‐values were significant, intercondition differences were subsequently tested with post hoc Tukey or Dunn's test, respectively. Comparison of TFA metrics (coherence, gain, phase) between all three recordings were performed at the frequency of squatting (0.05 Hz) but also at VLF range (Appendix S1; https://doi.org/10.25392/leicester.data.11860014 .v1). One‐way repeated measures ANOVA compared TFA metrics between recordings. Two‐way mixed ANOVA was used to assess differences between age group and SSM depth, as well as any interaction between the two. Exertion scores and SSM depth were compared between SSM_D_ and SSM_S_ using Student's *t* test and within age groups using paired *t* test. In the presence of significant *F*‐values from the ANOVA, post hoc pairwise comparisons were performed with Tukey's test. Intrarecording differences in EtCO_2_ were assessed with Friedman's and Wilcoxon tests. *p* < .05 was deemed as statistically significant.

## 3 RESULTS

Thirty‐four participants were recruited to the study. Of these, two were excluded due to inadequate bilateral temporal windows (both older group: one male, one female). All 32 participants included in the study performed the complete protocol with 15 maneuvers for both the deep and shallow SSM and none of the subjects reported any presyncopal symptoms. The demographics for the remaining 32 participants included in the analysis are presented in Table 1.

**Table 1:** Demographics by age group

	Older (n = 16)	Younger (n = 16)	Total (n = 32)
Sex			
Female	8	8	16
Handedness			
Right	14	15	29
Left	1	0	1
Ambidextrous	1	1	2
Smoking status			
Never	14	14	28
Ex‐smoker	2	1	3
Current smoker	0	1	1
Ethnicity			
White British	14	5	19
White Other	2	3	5
White and Asian	0	2	2
Indian	0	5	5
Asian other	0	1	1
Age (years)	57.1 ± 5.5	23.2 ± 3.4	40.3 ± 17.6
BMI (kg/m^2^)	24.5 ± 3.5	23.8 ± 3.4	24.2 ± 3.5

Note

Values of age and BMI are given as mean ± *SD*.

Abbreviation: BMI, body mass index.

### 3.1 Depth of SSM

The mean change in thigh angle during SSM_D_ and SSM_S_ was 67.4 ± 10.0 and 11.6 ± 8.7 degrees, respectively (*p* < .01). Participants achieved a mean depth of 13.7 ± 11.6 and 70.3 ± 7.2° from horizontal in the SSM_D_ and SSM_S_ positions, respectively (*p* < .01).

Differences in thigh angle for SSM_D_ and SSM_S_ were not statistically significant between age groups on Student's *t* test (*p* = .23 and *p* = .99, respectively).

### 3.2 Hemodynamic effects of SSM depth and age

**Figure 2:**
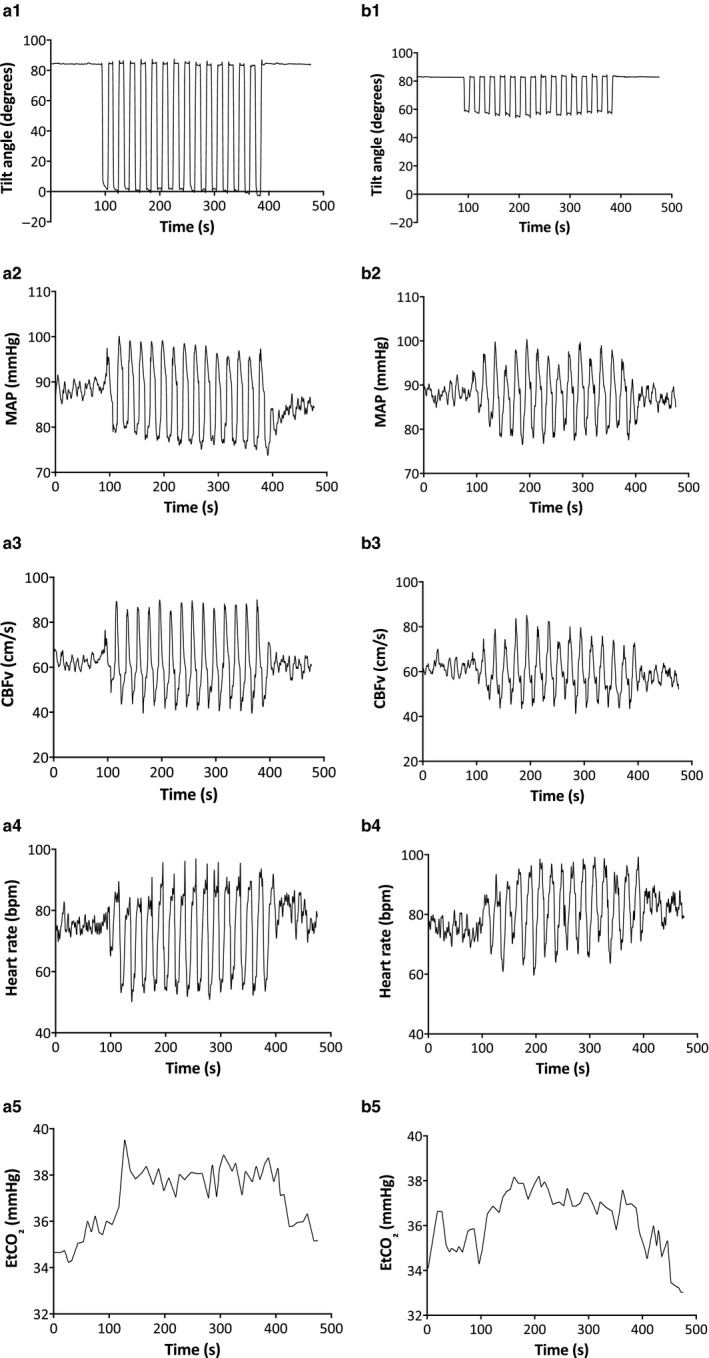
Time series of hemodynamic responses for SSM_D_ (a) and SSM_S_ (b) from an individual subject. Tilt angle expressed in degrees from horizontal (A1, B1); MAP (A2, B2); CBFv (A3, B3); Heart Rate (A4, B4); EtCO_2_ (A5, B5)

There were no differences between right and left MCA for any hemodynamic parameters, and values were therefore averaged across hemispheres for further analysis. EtCO_2_ (*p* = .02) and CBFv (*p* = .01) were lower in SSM_S_ compared to SSM_D_ (Table 2), but not different from baseline standing. The range of the MAP (ΔMAP) and CBFv (ΔCBFv) excursions during SSM_D_ was significantly higher (*p* < .001) than that for SSM_S_ (Table 2), without an effect of age. Temporal changes to SSM_D_ and SSM_S_ for a single participant are shown in Figure 2, demonstrating large changes in hemodynamic parameters in response to the squatting motion, as measured using the tilt sensor attached to the thigh. Intrarecording variability in EtCO_2_ was significantly larger for SSM_D_ (2.19 ± 0.78 mmHg; Friedman's, *p* = .00002), without differences between SSM_S_ (1.50 ± 0.55 mmHg) and baseline (1.25 ± 0.48 mmHg, *p* = .16). These differences in EtCO_2_ intrasubject variability were not influenced by age.

**Table 2:** Hemodynamic parameters by recording

Parameter	Standing	SSM_D_	SSM_S_	p‐value
CBFv MCA (cm/s)	59.1 ± 8.5^b^, ^c^	64.7 ± 10.4	61.6 ± 8.8^b^	<.01
MAP (mmHg)	92.6 ± 10.7^b^, ^c^	97.9 ± 12.4	98.3 ± 13.5	<.01
Systolic BP (mmHg)	121.4 ± 17.4^c^	130.6 ± 24.8	130.5 ± 24.6	<.01
Diastolic BP (mmHg)	80.6 ± 9.1	82.4 ± 9.7	84.2 ± 11.3	.01
HR (bpm)	78.7^c^ (73.5–88.7)	86.1 (79.1–93.9)	83.7 (77.7–98.7)	<.01
EtCO2 (mmHg)	35.5 ± 3.2^b^	38.3 ± 3.7	36.6 ± 3.6^b^	.01
ΔMAP younger group (mmHg)	—	30.55 ± 13.41	12.96 ± 8.40	<.001
ΔMAP older group (mmHg)	—	37.83 ± 18.45	22.21 ± 15.55	<.001
ΔCBFv younger group (cm/s)	—	39.70 ± 12.15	13.66 ± 4.94	<.001
ΔCBFv older group (cm/s)	—	38.97 ± 8.68	18.88 ± 12.83	<.001

Note

Normally distributed data are given as mean ± *SD*, non‐normally distributed data are given as median (IQR). *p*‐values were determined by repeated measured ANOVA and post hoc Tukey tests to compare between recordings. HR was compared using Friedman's test and Dunn's multiple comparison tests.

Abbreviations: BP, blood pressure; CBFv, cerebral blood flow velocity; EtCO_2_, end‐tidal CO_2_; HR, heart rate; MAP, mean arterial pressure; MCA, middle cerebral artery; ΔCBFv, range of CBFv excursion during SSM; ΔMAP, range of MAP excursion during SSM.

^b^ Reduced compared to SSM_D._

^c^ Reduced compared to SSM_S_.

**Figure 3:**
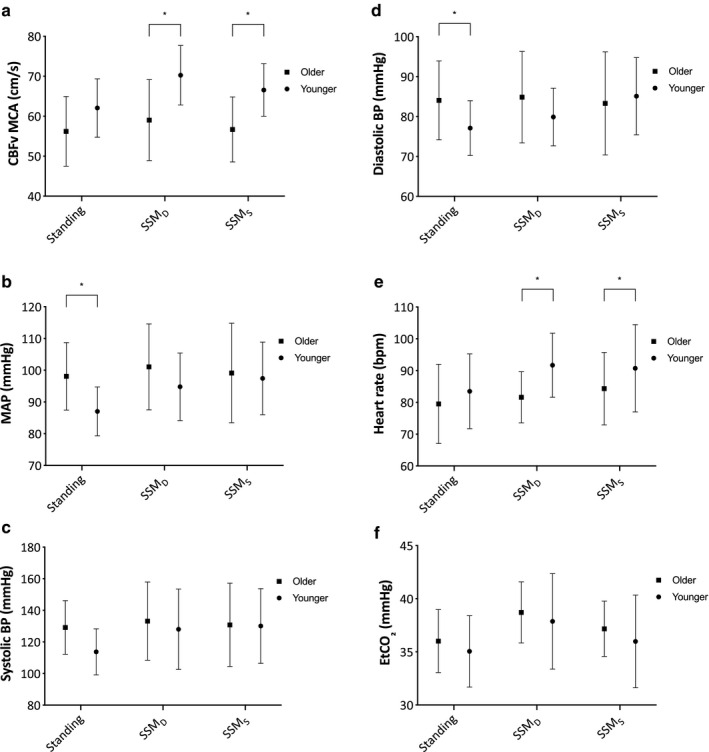
Mean CBFv (a), MAP (b), Systolic BP (c), Diastolic BP (d), Heart rate (e), and EtCO_2_ (f) for each recording in the older (squares) and younger (circles) groups. Error bars represent *SD*. *p < .05

Mean CBFv was not significantly different between the age groups at baseline, however, MAP and diastolic BP were higher in the older age group (both *p* < .01). During performance of SSM, CBFv (*p* < .01) and HR (*p* = .02) were higher in the younger age group (Figure 3).

### 3.3 Dynamic cerebral autoregulation

All 32 participants were included in the final analysis. Repeated measures ANOVA *p*‐values are given in Table 3. ARI was lower in the SSM_D_ position compared to baseline standing (post hoc *p* = .0002) and SSM_S_ (post hoc *p* < .0002), without differences between SSM_s_ and baseline standing. At the driven frequency of 0.05 Hz, coherence was higher in both SSM_D_ and SSM_S_ compared to baseline standing (both *p* < .0002), and in SSM_D_ compared to SSM_S_ (post hoc *p* = .003). Gain was higher in SSM_D_ compared to baseline standing (*p* < .001) and SSM_s_ (*p* = .005), but SSM_s_ was not different from baseline standing. Phase was lower in SSM_D_ compared to baseline standing (post hoc *p* = .028) and SSM_S_ (post hoc *p* < .0005) (Table 3). Averaging spectral estimates for the VLF band, instead of punctual values at 0.05 Hz, led to very similar results (Appendix S1; https://doi.org/10.25392/leicester.data.11860014 .v1).

**Table 3:** Transfer function analysis parameters and autoregulation index by recording

Parameter	Standing	SSM_D_	SSM_S_	p‐value
ARI	6.03 (5.20–6.49)	4.46^a^ (4.03–5.22)	5.96 (5.40–6.69)	<.01
Coherence	0.557 (0.274–0.714)	0.987 (0.977–0.993)	0.970^b^ (0.912–0.988)	<.001
Gain (%/%)	1.16 (0.89–1.40)	1.55^c^ (1.27–1.92)	1.46^c^ (1.24–2.02)	<.001
Phase (radians)	0.87 (0.72–1.14)	0.64^a^ (0.51–0.71)	0.94 (0.76–1.16)	<.001
BP power (mmHg)^2^/Hz	64.18 (24.31–127.17)	5218.5^a^ (2752.1–12973.3)	1453.7^b^ (444.8–2800.4)	<.001
CBFv power (cm/s)^2^/Hz	58.76 (39.32–94.83)	6802.9^c^ (4812.3–10924.4)	1110.2^b^ (404.6–2281.1)	<.001

Note

Values are given as median (IQR). TFA and power spectral parameters were extracted at the frequency of squatting (0.05 Hz). *p*‐value determined by repeated measure Friedman test. Paired comparisons by Wilcoxon signed‐rank test.

Abbreviation: ARI, autoregulation index.

^a^ *p* < .01 compared to baseline standing and SSM_S_

^b^ *p* < .01 compared to both baseline standing and SSM_D_

^c^ *p* < .01 compared to baseline standing.

ARI was lower during SSM_D_ compared to SSM_S_ for both age groups and was significantly lower in the older compared to younger age group (*p* = .03) with no depth interaction (*p* = .99). Coherence was lower during SSM_S_ compared to SSM_D_ and was lower in the younger compared to older age group (*p* < .01) with a significant interaction (*p* < .01). Gain showed no significant difference between age groups (*p* = .14) nor SSM depth (*p* = .46), however, phase was significantly higher in the younger age group (*p* < .01) and during SSM_S_ (*p* < .01) with a significant interaction (*p* < .01) (Table 4).

**Table 4:** TFA parameters between SSM depth split by age group

Parameter	Older (n = 16)		Younger (n = 16)		p‐values		
	SSM_D_	SSM_S_	SSM_D_	SSM_S_	Depth	Age	Interaction
ARI	4.42 ± 0.84	5.73 ± 0.77	4.90 ± 0.77	6.20 ± 0.72	<.01	.03	.99
Coherence	0.990 ± 0.006	0.975 ± 0.036	0.979 ± 0.010	0.856 ± 0.144	<.01	<.01	<.01
Gain (%/%)	1.90 ± 0.74	1.62 ± 0.50	1.45 ± 0.33	1.55 ± 0.68	.46	.14	.10
Phase (radians)	0.57 ± 0.15	0.80 ± 0.22	0.66 ± 0.13	1.11 ± 0.22	<.01	<.01	<.01

Note

Data are given as mean ± *SD*. Coherence, gain, and phase values extracted for the 0.05 Hz harmonic. *p*‐values from two‐way mixed ANOVA

Abbreviations: ARI, autoregulation index; SSM_D_, deep squat‐stand maneuver; SSM_S_, shallow squat‐stand maneuver.

### 3.4 Exertion scores

Mean exertion scores for SSM_D_ and SSM_S_ across the whole cohort were 3.8 ± 1.8 versus 3.8 ± 1.8 (*p* = .89). Participants in the older age group found SSM_S_ significantly more effortful than the SSM_D_ (3.8 ± 1.7 vs. 3.0 ± 1.6, *p* < .01). Conversely, the younger age group found the SSM_D_ significantly more difficult than SSM_S_ (4.5 ± 1.7 vs. 3.8 ± 1.8, *p* < .01).

## 4 DISCUSSION

### 4.1 Main findings

In agreement with previous studies, SSM at both maximal and shallower depths produced low‐frequency coherence values significantly higher than spontaneous BP oscillations at rest. SSM_D_ produced lower estimates of ARI and phase compared to SSM_S_, which were more pronounced in the older age group. Participants in the older age group found SSM_D_ significantly less effortful than SSM_S_.

These findings suggest that both deep and shallow SSM lead to more robust estimates of dCA parameters than spontaneous BP oscillations. They also indicate that CA may be less efficient during deep SSMs, which is attenuated by younger age. Shallower SSM could therefore be seen as a more promising approach to dCA measurement in older populations, however, they were deemed less tolerable than the deep alternative. As discussed in the following section, differences in ARI and other spectral parameters, between deep and shallow SSM, might be due to differences in partial pressure of arterial CO_2_ (PaCO_2_) resulting from different patterns of breathing associated with these maneuvers.

### 4.2 Methodological developments

Coherence was significantly higher for SSM_D_ than for SSM_S_ (Table 3) although both produced coherence values far above the 95% confidence limit deemed sufficient for CA measurement, and were significantly higher than baseline (Claassen et al., 2016). This implies that SSM at both depths can be used to measure CA more effectively than spontaneous BP oscillations. ARI was lower during SSM_D_ compared to baseline recordings and SSM_S_, which was consistent with the TFA comparisons between recordings. These findings suggest a less efficient autoregulatory response during SSM_D_ compared with SSM_S_. If SSM_D_ were to be used in clinical assessment, it may be difficult to determine whether a low CA value was due to underlying pathophysiology, or the SSM_D_ itself.

One possible explanation for these findings is the relative hypercapnia induced by SSM_D_ which has notably been reported previously (Barnes et al., 2017). This would be expected to increase coherence and to depress dCA. However, a previous study reported decreasing ARI and increasing EtCO_2_ with increasing number of SSM at around 24° from horizontal, without effect on coherence values (Barnes et al., 2018).

Conversely, although EtCO_2_ during SSM_S_ seems to suggest relative hypocapnia, when compared to SSM_D_, in fact EtCO_2_ was not significantly different from baseline resting during SSM_S_. The physiological reasons for this are not entirely clear. Standing can induce a ventilation/cardiac output mismatch that might explain the lower EtCO_2_ during the standing baseline recording, as compared to supine rest (Immink et al., 2006; Serrador, Hughson, Kowalchuk, Bondar, & Gelb, 2005). Although subjects were asked to breathe normally and try to avoid holding their breath, similarly to a Valsalva maneuver, inevitably the squat‐stand exercise leads to changes in breathing patterns, and it is not unreasonable to suggest that these would be more pronounced during SSM_D_ as compared to SSM_S_, thus explaining the relative hypercapnia of the former. This interpretation is also supported by the increased variability in EtCO_2_ within recordings that was also more pronounced during SSM_D_, in comparison with baseline and SSM_S_.

It is likely that the physiology behind BP changes differs between SSM depths. During SSM_S_, there is a maintained contraction of the quadriceps and gluteal muscles throughout the entire 10‐second squat. This minimizes venous pooling and increases blood return to the thoracic circulation, thus increasing cardiac output during the squat phase (Krediet et al., 2005, 2007). Muscle contraction also initiates a transient peripheral ischemia upon squatting, which enhances vasodilation upon standing, causing a reduction in total peripheral resistance and subsequent drop in BP (Tanaka, Sjöberg, & Thulesius, 1996). Krediet et al demonstrated that lower‐body muscle tensing can attenuate the transient BP decrease after standing up from squat, and Zhang et al used a pulley system to show reduced BP oscillations during passive SSM, whereby the muscle reflex and central command during active SSM were less of a factor (Krediet et al., 2007; Zhang et al., 2009). When returning to the standing position, BP drops due to vasodilatation of the lower limb vessels and subsequent drop in total peripheral resistance (Tanaka et al., 1996). These changes will be attenuated for SSM_S_ as subjects start their return to standing from a semi‐squatting position. Exploration into the effect of lower‐body muscle tensing on hemodynamics and CA measurement could provide an alternative to SSM in bed‐bound patients, with potentially increased tolerability compared to the uncomfortable thigh‐cuff maneuver (Sorond, Serrador, Jones, Shaffer, & Lipsitz, 2009). With different depths of squatting, the overall change in BP with each maneuver (ΔMAP) was larger with SSM_D_ in comparison with SSM_S_ (Table 2) as also indicated by the differences in spectral power observed between the two modalities (Table 3). Further work is needed to assess whether the larger excursion in BP with SSM_D_ could contribute to explain the lower ARI values observed during this maneuver, when compared to SSM_S_. On the other hand, this possibility becomes less likely, when one compares the much larger differences in spectral power between SSM_S_ and baseline (Table 3), and note that there were no differences in ARI between the two conditions.

SSM_D_ involved participants reaching maximal comfortable depth. This generally involved lifting their heels off the ground for enhanced comfort, allowing them to more easily tolerate the squat position. This technique may have reduced muscle contraction throughout the squat phase requiring effort to obtain the squat position and return to standing. Therefore, it is suggested that the BP changes during SSM_D_ are most likely from compression of the veins in the leg, and the effect of gravity on the peripheral circulation by bringing the feet closer to the heart, rather than the effects of the muscle mechanoreflex (O’Donnell & McIlroy, 1962).

Interestingly, there was a larger increase in CBFv during SSM_D_ in the younger group than SSM_S_, despite almost identical changes in BP and HR during both SSM recordings. This may suggest that the drastic change in posture during SSM_D_ has more of an impact on CBFv than the hemodynamic effects of the SSM. Cardiorespiratory fitness has been shown to increase TFA gain derived from standard SSM (Labrecque et al., 2017), but this was only observed at a squatting frequency of 0.10 Hz. In our study, no differences in gain were observed between the young and older groups, but further work is needed to clarify whether differences in cardiorespiratory fitness could explain the differences we found in ARI and phase between the two age groups.

To our knowledge, this is the first time exertion scores between depths of SSM have been collected and compared between age groups. When analyzed by age group, SSM_D_ were found to be significantly more effortful in the younger participants, and SSM_S_ were deemed significantly more effortful in the older age group. These findings defy our hypothesis that SSM_S_ are less effortful than SSM_D_ and area very relevant finding for the clinical applicability of SSM.

### 4.3 Clinical implications

CA has been shown to be affected in disease states such as stroke (Xiong et al., 2017), Alzheimer's disease (Claassen, Diaz‐Arrastia, et al., 2009) and traumatic brain injury (Bor‐Seng‐Shu et al., 2012). Additionally, impaired CA postischemic stroke has been demonstrated as an independent predictor of outcome and secondary complications, such as hemorrhagic transformation and cerebral oedema (Castro, Azevedo, & Sorond, 2018; Castro, Serrador, Rocha, Sorond, & Azevedo, 2017; Chi et al., 2018). Therefore, individualized hemodynamic management may be a key to improving clinical outcomes in stroke patients (Castro et al., 2018).

In order for CA to be measured effectively in these populations, we must first determine whether different protocols are feasible in the age groups in which these conditions are most prevalent. Results from studies conducted on young, healthy participants are not easily generalizable to older, comorbid populations. This study increased clinical applicability by recruiting cohorts of both younger and older participants, to test feasibility across age groups and to determine any age‐related changes in CA during the performance of SSM. As SSM represent an orthostatic challenge which is frequently encountered in daily life, for example when bending down to pick something off the floor or tie a shoelace, any evidence to suggest CA impairment under these circumstances increases our awareness of high‐risk situations for significantly altered CBF.

Literature surrounding CA in healthy aging has largely shown an intact mechanism when measured using spontaneous BP oscillations (Yam et al., 2005), steady‐state cycling (Fisher, Ogoh, Young, Raven, & Fadel, 2008), respiratory maneuvers (Dineen, Panerai, Brodie, & Robinson, 2011), and sit‐stand maneuvers (Xing et al., 2017). From this study, the similar ARI and phase values at baseline between age groups support the literature stating that CA is unaffected by aging during spontaneous BP oscillations at rest (Yam et al., 2005). These results contradict previous evidence to suggest CA function is maintained with age during BP manipulation (Carey, Eames, Blake, Panerai, & Potter, 2000; Oudegeest‐Sander et al., 2014; Sorond et al., 2009). This is the first study to measure ARI during repeated SSM and compare between two age groups and is therefore, a very relevant and novel finding. This may demonstrate that repeated SSM provide a unique challenge to the autoregulatory response that separates the capabilities of CA between age groups. Further exploration of these differences is warranted with the use of time‐domain analysis to explore the effect of individual covariates, such as arterial compliance, on the CBFv response.

The only physiological differences we detected between age groups at baseline were a higher MAP, and lower CBFv, in the older group. These effects of aging are well documented in the literature and equate to an increased cerebrovascular resistance index, calculated as the ratio between MAP and CBFv, potentially caused by thickening and stiffening of arterial walls with increasing age (Oudegeest‐Sander et al., 2014; Xing et al., 2017).

During performance of SSM, the difference in CBFv between age groups widened further to a maximum of 12 cm/s during SSM_D_. HR was also significantly increased in the younger compared to the older group during the SSM, despite resting HR between the groups starting at a similar level. This phenomenon is in keeping with the known effects of aging on the arterial‐cardiac baroreflex function during repeated SSM (Zhang et al., 2009).

### 4.4 Study limitations

A general limitation of TCD is the use of CBFv as a surrogate measurement of CBF. A recent study showed that there was no significant change in MCA diameter provided PaCO_2_ levels remained within a range of 7.5 mmHg either side of normocapnia, above which led to underestimation of the actual CBF (Verbree et al., 2014). Mean EtCO_2_ values in this study were within a range of 2.8 mmHg and were therefore unlikely to have had any effect on CBF.

During SSM_D_, participants were instructed to adopt the deepest squat position that they could comfortably achieve, which highlighted two variants in methodology: heels off the ground, and feet flat to the floor. Although only a minority of participants in the younger group in this study adopted the latter, it could have had a significant impact on our results and undoubtedly influenced the exertion scores for these participants. It was unlikely that all participants would have been able to comply with the SSM with feet flat to floor, as this likely poses a considerably more difficult physical challenge. Therefore, the majority of participants, including all those in the older group, chose to perform SSM_D_ with heels off the floor. It would be of considerable interest to study the hemodynamic effects and exertion scores between these two SSM_D_ techniques. On the other hand, due to the relatively small number of participants in each group, we decided against randomizing the order of the deep and shallow maneuvers, SSM_D_ was always performed first. Therefore, it is possible that older participants were more fatigued than younger ones and this could have influenced their assessment of exertion. Nevertheless, all participants were allowed to rest for as long as needed between the two sets of maneuvers.

During aerobic exercise, brain activation causes an increase in CBF in order to deliver oxygen to the motor areas involved in carrying out physical tasks (Ogoh & Ainslie, 2009; Secher, Seifert, & Van Lieshout, 2008). It is possible this could have had a small effect on regional CBF, however, it is unlikely these changes in CBF would follow the frequencies of SSM and they would in theory be relatively much smaller than the BP changes induced by the SSM (Claassen, Diaz‐Arrastia, et al., 2009). Regional differences in CBF could also influence the differences observed between SSM_S_ and SSM_D_ in the MCA and for this reason our results cannot be generalized to other intracerebral arteries such as the posterior or anterior cerebral arteries.

Ogoh et al have shown that CA was impaired during exhaustive exercise (Ogoh et al., 2005). A proposed explanation for this is the development of an acute hyperammonemia, which has been linked to impaired sympathetic regulation of CBF in patients with acute liver failure, during exhaustive exercise (Lagi et al., 2002). Although this offers an appealing explanation to the reduced ARI during SSM_D_, the subjective measure of exertion during the SSM suggests participants in this study were far from exhausted.

We asked participants to maintain their breathing as close to normal as possible and to avoid performing a Valsalva maneuver during the squatting phase. The similar values of mean EtCO_2_ observed for SSM_S_ and baseline, as well as the similar intrarecording variability of EtCO_2_ in these two conditions, suggest that breathing was not altered by the shallow maneuver compared to standing at rest. On the other hand, both relative hypercapnia, and increased variability of EtCO_2_ intrarecording, took place during SSM_D_ which will require consideration in future studies. We are not aware of previous studies in the literature where an EtCO_2_ clamping system has been adopted. Such a system would be needed for rigorous control of PaCO_2_, entailing expensive equipment to clamp EtCO_2_ including the use of a mask. The use of the mask, and the changes in breathing required to keep EtCO_2_ at a clamped level, would alter normal physiology in a number of different ways. Secondly, giving participants visual feedback about their breath‐by‐breath EtCO_2_, and asking them to alter their breathing in a way to keep it around normocapnic values, would lead to considerable cognitive stimulation that would behave as “noise,” by means of changes in CBFV due to the mechanism of neurovascular coupling.

All participants were able to complete all 15 SSM at each depth. Although this demonstrates the feasibility of SSM in our population, this may not be generalizable to a patient population, as SSM may not be as acceptable to the older‐old, obese, or patients with comorbidities. Participants were generally fit and well, with an average BMI of 24.3 ± 3.6 kg/m^2^ and were free from any musculoskeletal difficulties. Additionally, SSM are not appropriate for bed‐bound or severely mobility restricted patients, further limiting their clinical applicability.

The tilt sensor measured the change in thigh angle compared to horizontal, rather than the degree of flexion of the knee, hence why SSM_S_ demonstrated a tilt angle much less than the 45 degrees that was manually measured in all subjects. Analysis of SSM depth could be improved by determining the net joint movement and muscular torque generated by each muscle group, which can be used to calculate relative muscular effort (Bryanton, Kennedy, Carey, & Chiu, 2012). This would also remove the subjectivity of the exertion scores.

Finally, subjects may have rested against the bed that was set behind them as a guide during SSM_S_, which could have potentially reduced the muscular effort that is required to maintain the squat position. However, participants are unlikely to have done so given the reported exertion scores for SSM_S_.

### 4.5 Future work

This study has highlighted some fascinating differences between depths of SSM in the context of measuring CA. Further analyses of the individual covariates involved in CBF control, and their influences on CBFv during differing depths of SSM, are now needed in order to fully decipher the reasons for these discrepancies. In particular, larger sample sizes are needed to assess the influence of sex and its potential interaction effects with aging and depth of squatting.

We have shown the value of SSM for improving measurements of CA in a healthy population. The next logical step is to assess the feasibility of SSM in disease states, such as acute stroke and transient ischemic attack (TIA). If SSM are proven to be feasible in these populations, we could advance the knowledge of CA deficits suggested by studies using spontaneous BP oscillations. There have been very few studies assessing the acute effect of TIA on CA, as most studies have focused on stroke populations. Individuals who might present early deterioration in dynamic CA, mainly during changes in posture, could benefit from tighter control of BP. Additionally, it may be an important predictor of future cerebrovascular events in this already higher risk population.

This study has shown the effects on CA of an “older” group of healthy participants, though in reality this group is more likely to be classified as middle‐aged with a mean age of 57 years. It would be of considerable benefit to apply SSM to a cohort of participants in the “older old” category. It would be expected that the compliance of SSM with increasing age would decrease due to various comorbidities, most notably musculoskeletal conditions, as was experienced in previous studies attempting SSM in older populations (Oudegeest‐Sander et al., 2014; Zhang et al., 2009).

In the classical SSM_D_, it has been demonstrated that estimates of dynamic CA show greater efficiency during squatting, when compared with the standing phase (Brassard et al., 2017; Panerai et al., 2018). Whether this phenomenon of “hysteresis” (Brassard et al., 2017) or “directional sensitivity” (Panerai et al., 2018) also takes place during SSM_S_ would warrant further investigation, as it could be an additional reason to give preference to the shallow modality, due to less complex assessment techniques required in the presence of hysteresis.

## 5 CONCLUSION

This study has demonstrated a number of novel and exciting findings to add to the field of CA measurement with the use of SSM. SSM_D_ produced excellent coherence values and were more tolerable to an older group of healthy participants, however, they produced ARI and TFA estimates that indicated less efficient CA at this depth. On the other hand, SSM_S_ also produced improved coherence values as compared to spontaneous BP oscillations and did not affect estimates of CA, however, were deemed as more effortful to a group of healthy individuals with a mean age of 57 years. Additionally, compared to a younger group of participants, the older group had less efficient CA responses during performance of SSM at varying depths.

Further studies are now warranted to explore the reasons for these physiological and age‐group differences, and to refine methodological standards by determining the most effective SSM depth for assessing dCA. It is hoped the results from this study will further ongoing research in this field, specifically in patient populations and those previously excluded from studies of dCA.

## CONFLICT OF INTEREST

The authors declare that there is no conflict of interest.

## AUTHORS’ CONTRIBUTIONS

V. J. H, R. B. P, and T. G. R conceived and designed research; A. ﻿P. B carried out experiments; A. P. B and R. B. P analyzed data; A. B. P, V. J. H, and R. B. P interpreted the experimental results; A. P. B drafted manuscript and prepared figures; A. P. B, V. J. H, R. B. P, and T. G. R edited and revised manuscript; V. J. H, R. B. P, and T. G. R approved final version of manuscript.

## Supporting information



Appendix S1
