# Supplementary material for: Does depth of squat‐stand maneuver affect estimates of dynamic cerebral autoregulation?
Source: Physiol Rep. 2020 Aug 18;8(16):e14549. doi: 10.14814/phy2.14549 (PMC7435029; doi:10.14814/phy2.14549)
Supplement: Supplementary file 1 — Appendix S1 [file PHY2-8-e14549-s001.pdf]

Does depth of squat-stand maneuver affect estimates of dynamic cerebral autoregulation?

Supplementary appendix

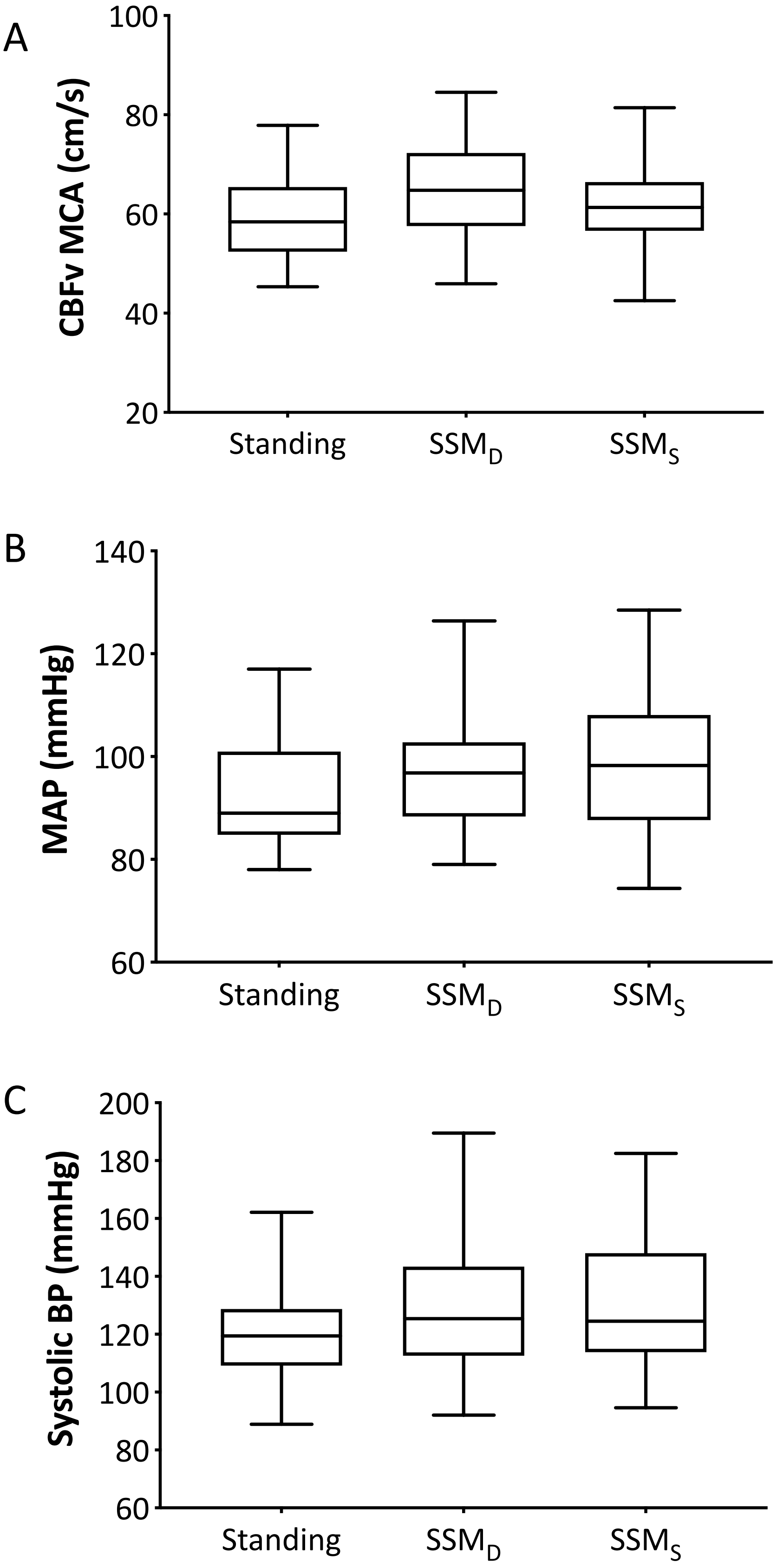

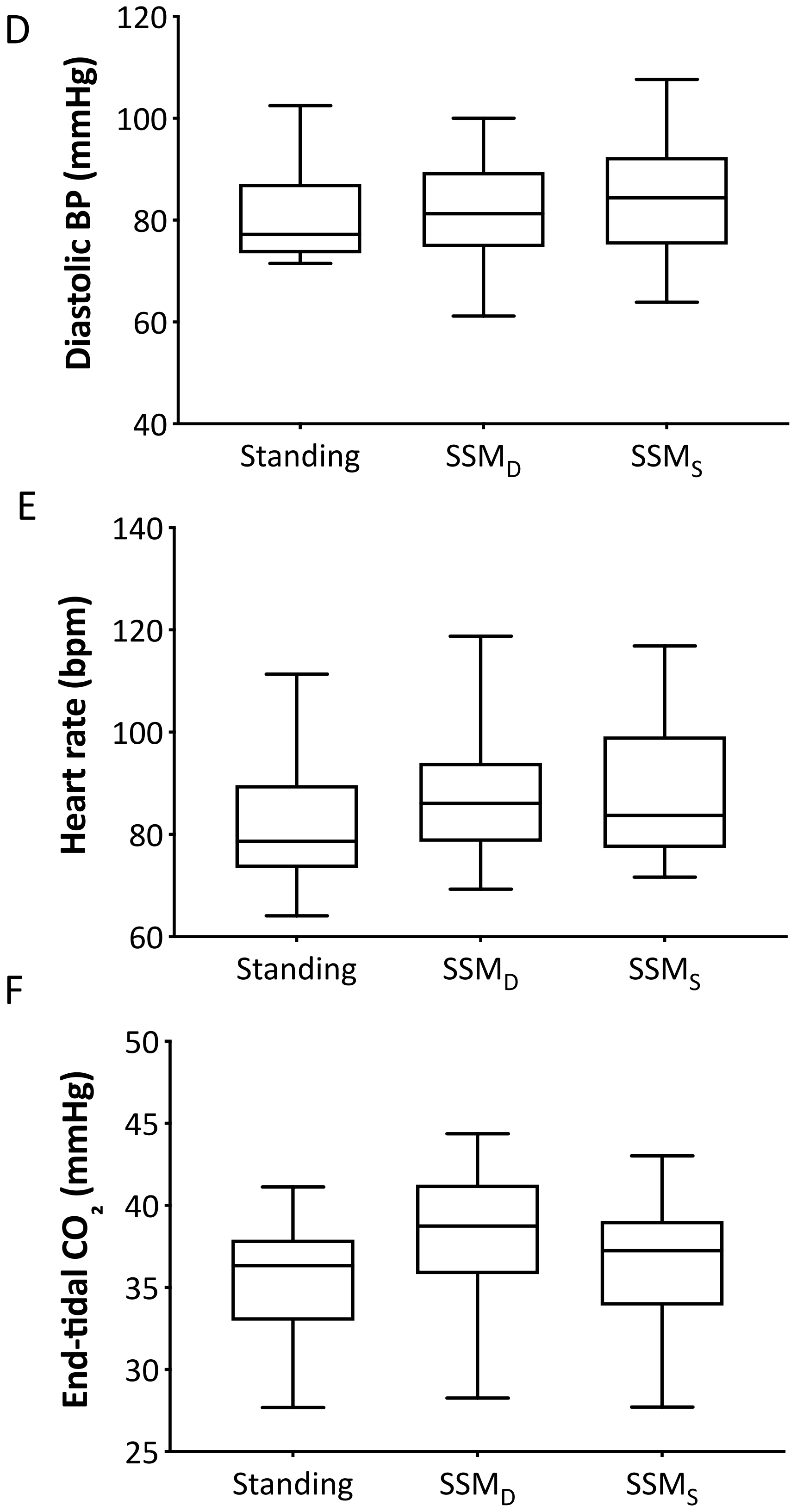

Figure S1: CBFv (A), MAP (B), Systolic BP (C), Diastolic BP (D), Heart rate (E) and $EtCO_2$ (F) for standing baseline, SSM_D and SSM_S. Box represents median and upper and lower IQR, whiskers represent maximum and minimum.

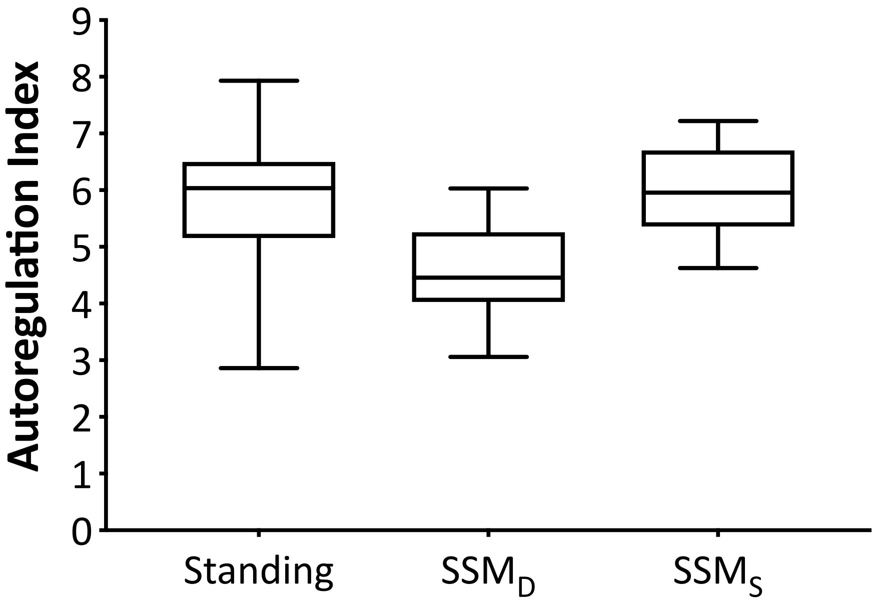

Figure S2: Autoregulation index for standing baseline, SSM_D and SSM_S

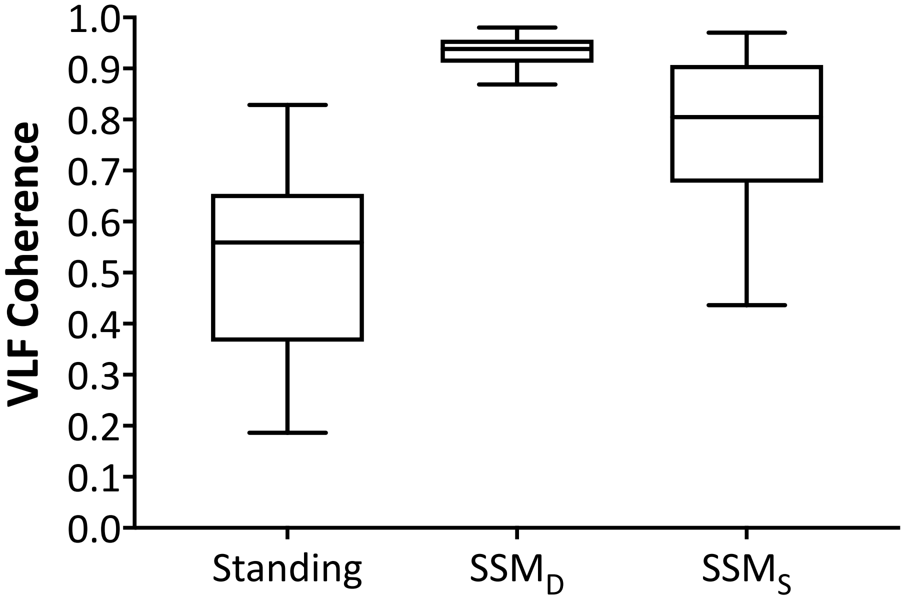

Figure S3: VLF coherence for standing baseline, SSM_D and SSM_S

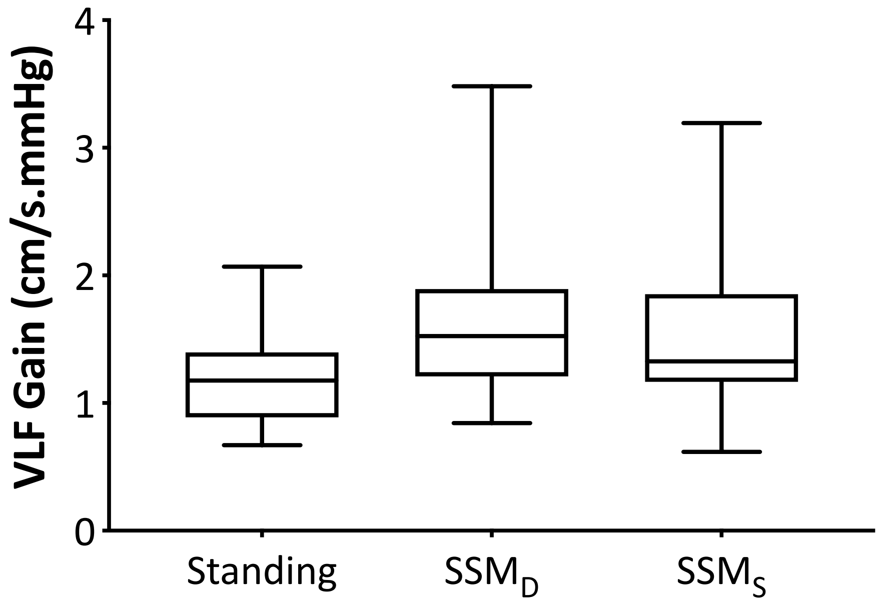

Figure S4: VLF gain for standing baseline, SSM_D and SSM_S

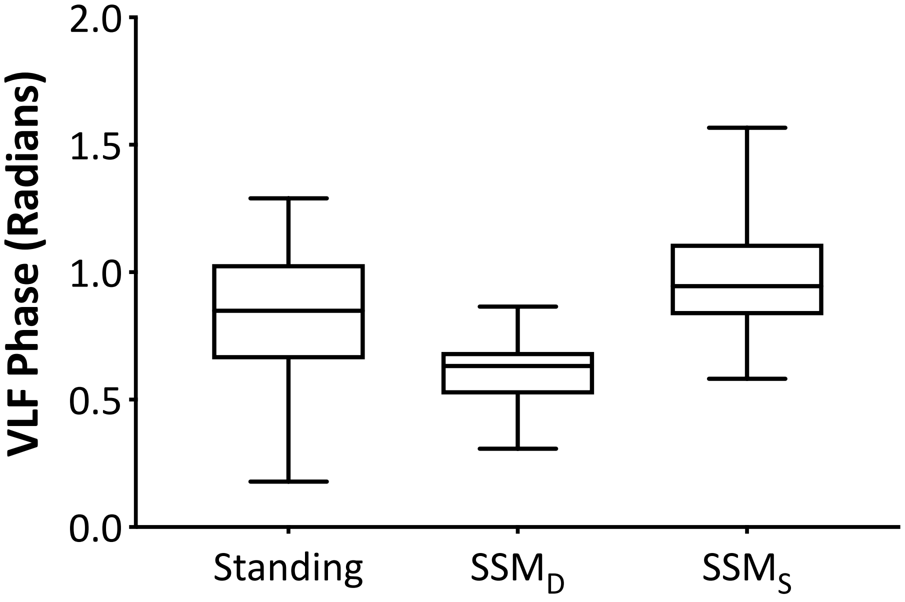

Figure S5: VLF phase for standing baseline, SSM_D and SSM_S

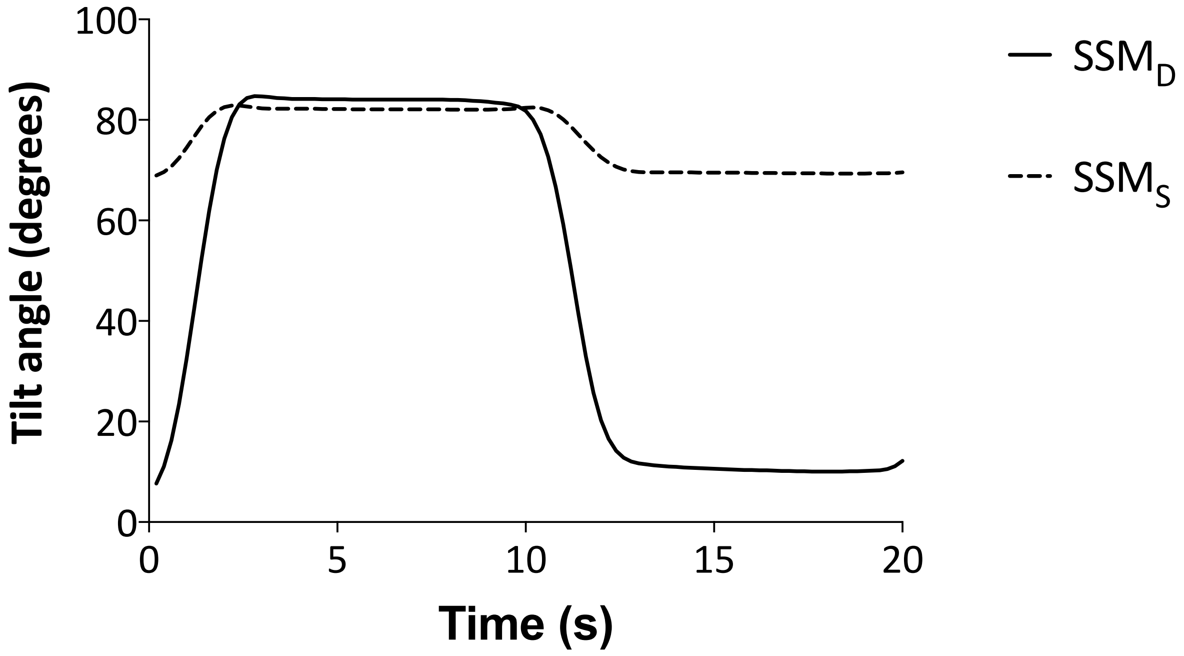

Figure S6: Average tilt angle from horizontal for SSM_D (solid) and SSM_S (dashed). Standing from squat was initiated at 0 seconds, subsequent squat was initiated at 10 seconds.

Table S1: Hemodynamic parameters according to SSM depth and age group

Parameter	Older (n=16)		Younger (n=16)		P-values	
	SSM_D	SSM_S	SSM_D	SSM_S	Age	Interaction
CBFv MCA (cm/s)	59.0 ± 10.1	56.7 ± 8.1	70.3 ± 7.4	66.6 ± 6.6	<0.01	0.47
MAP (mmHg)	101.1 ± 13.6	99.1 ± 15.7	94.8 ± 10.7	97.4 ± 11.4	0.36	0.19
Systolic BP (mmHg)	133.2 ± 24.8	130.8 ± 26.4	128.0 ± 25.5	130.1 ± 23.6	0.73	0.45
Diastolic BP (mmHg)	84.9 ± 11.5	83.3 ± 12.9	79.9 ± 7.2	85.1 ± 9.7	0.64	0.05
Heart Rate (bpm)	81.6 ± 8.0	84.3 ± 11.4	91.7 ± 10.1	90.7 ± 13.7	0.02	0.34
$EtCO_2$ (mmHg)	38.7 ± 2.9	37.2 ± 2.6	37.9 ± 4.5	36.0 ± 4.4	0.40	0.76

Values are given as mean ± SD. CBFv, Cerebral blood flow velocity; MCA, Middle cerebral artery; MAP, Mean arterial pressure; BP, Blood pressure; $EtCO_2$, End-tidal CO_2. P-values from two-way mixed ANOVA demonstrate the between-subject effects of age group on each hemodynamic parameter and the interaction between age group and depth of SSM.

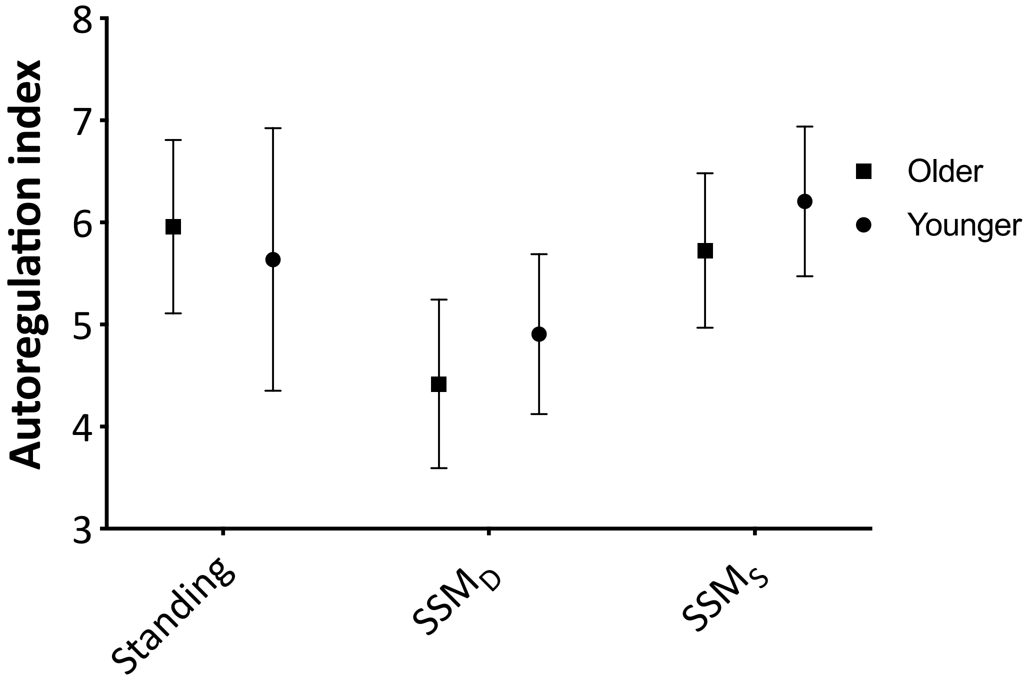

Figure S7: Autoregulation index split by younger (circles) and older (squares) age groups, during each baseline position and SSM depth. Error bars give SD.

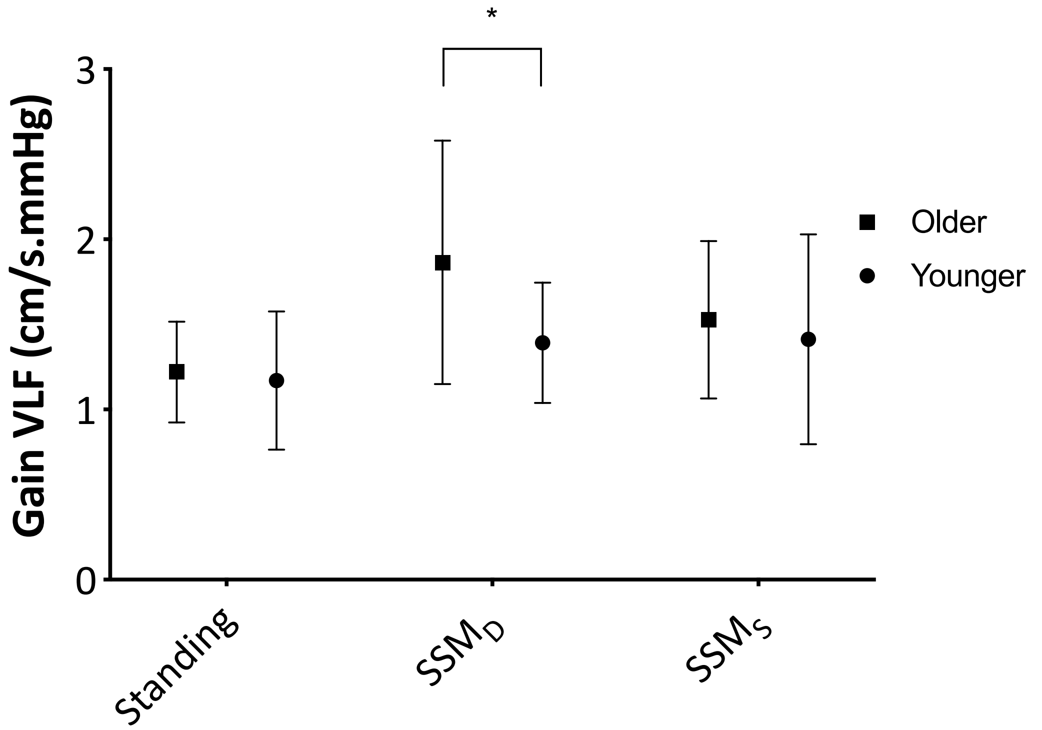

Figure S8: VLF gain split by younger (circles) and older (squares) age groups, during each baseline position and SSM depth. Error bars give SD. P-values are from student's t-test * = p<0.05

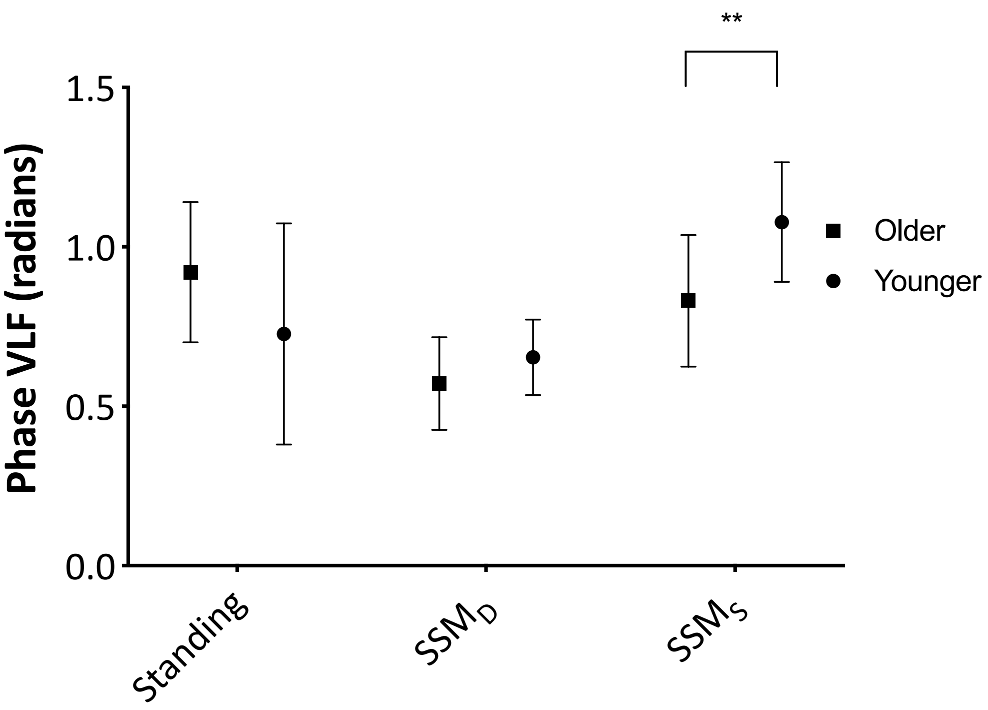

Figure S9: VLF phase split by younger (circles) and older (squares) age groups, during each baseline position and SSM depth. Error bars give SD. P-values are from student's t-test ** = p<0.01

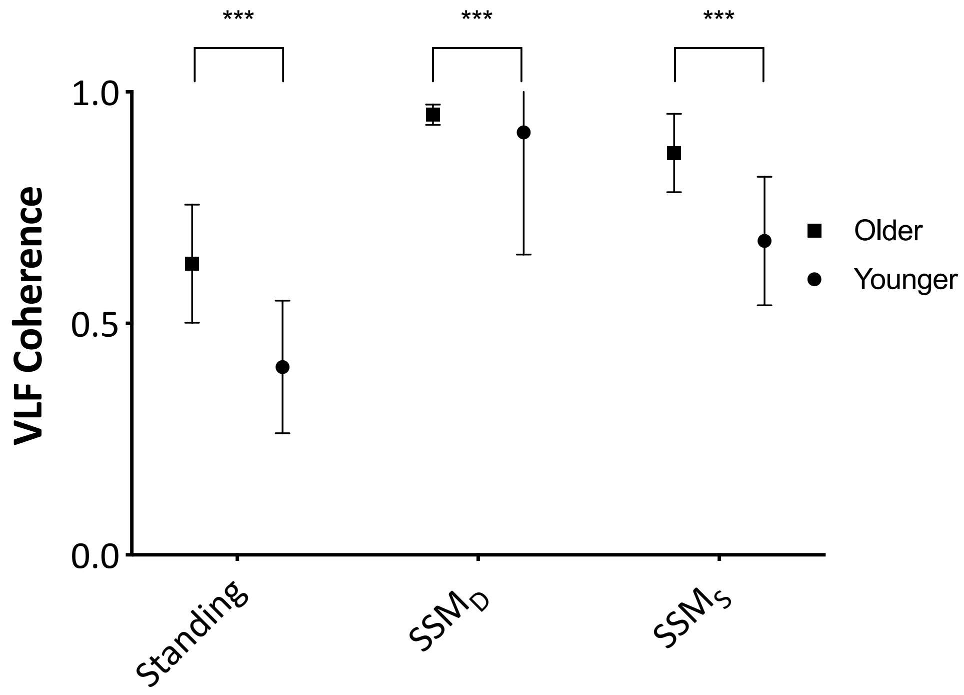

Figure S10: VLF coherence split by younger (circles) and older (squares) age groups, during each baseline position and SSM depth. Error bars give SD. P-values are from student's t-test *** = p<0.001.

Table S2: Mean change in thigh angle during SSMs between age groups

	Older	Younger	P-value
SSM_D	65.3 ± 9.2	69.5 ± 10.5	0.23
SSM_S	11.6 ± 9.1	11.6 ± 8.4	0.99

Values are given as mean ± SD. P-values are from Student's t-test.

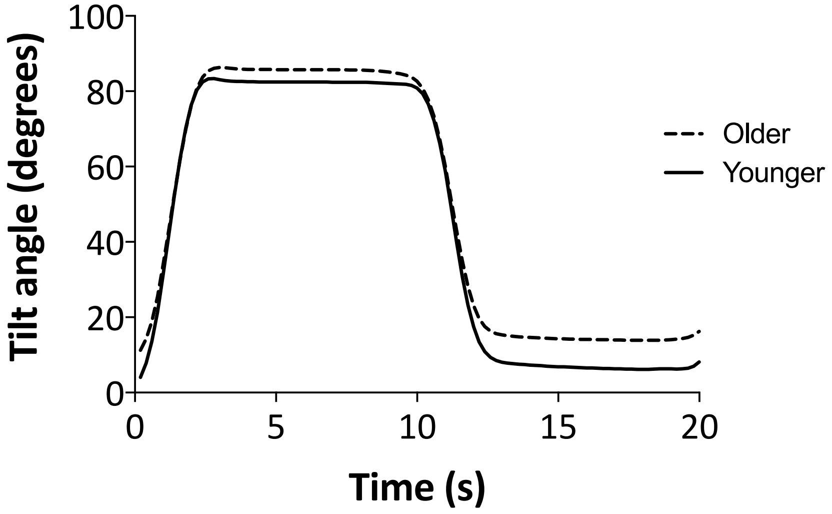

Figure S11: Mean SSM_D depth for younger (solid) and older (dashed) age groups. Tilt angle is given in degrees from horizontal.

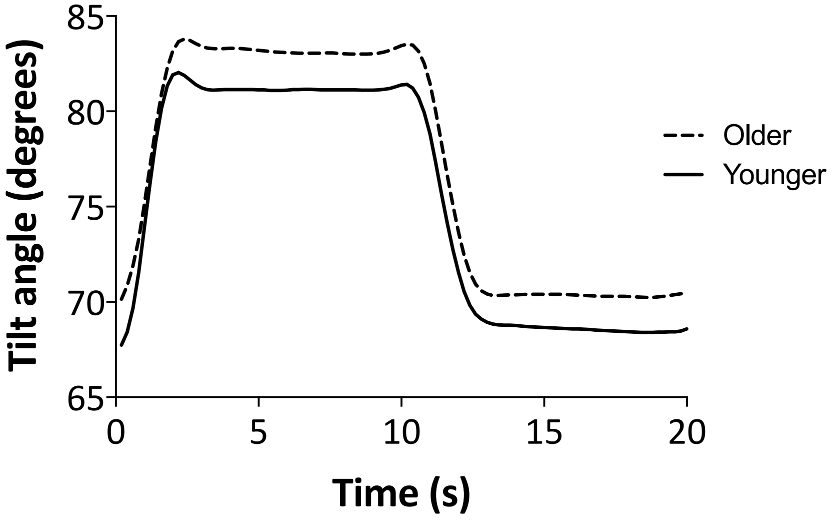

Figure S12: Mean SSM_S depth for younger (solid) and older (dashed) age groups. Tilt angle is given in degrees from horizontal. Note difference in vertical scale from Fig. S11.
